# Tumor necrosis factor-alpha promotes survival in methotrexate-exposed macrophages by an NF-kappaB-dependent pathway

**DOI:** 10.1186/ar3248

**Published:** 2011-02-15

**Authors:** Susan ZY Lo, James H Steer, David A Joyce

**Affiliations:** 1 Pharmacology Unit, School of Medicine and Pharmacology, University of Western Australia, 35 Stirling Highway, Crawley, Western Australia 6009, Australia

## Abstract

**Introduction:**

Methotrexate (MTX) induces macrophage apoptosis *in vitro*, but there is not much evidence for increased synovial macrophage apoptosis in MTX-treated patients. Macrophage apoptosis is reported, however, during clinical response to anti-tumor necrosis factor-alpha (TNF-α) treatments. This implies that TNF-α promotes macrophage survival and suggests that TNF-α may protect against MTX-induced apoptosis. We, therefore, investigated this proposal and the macrophage signaling pathways underlying it.

**Methods:**

Caspase-3 activity, annexin-V binding/7-aminoactinomycin D (7-AAD) exclusion and cell-cycle analysis were used to measure steps in apoptosis of primary murine macrophages and cells of the RAW_264.7 _macrophage cell line that had been exposed to clinically-relevant concentrations of MTX and TNF-α.

**Results:**

MTX induces apoptosis in primary murine macrophages at concentrations as low as 100 nM *in vitro*. TNF-α, which has a context-dependent ability to increase or to suppress apoptosis, efficiently suppresses MTX-induced macrophage apoptosis. This depends on NF-κB signaling, initiated through TNF Receptor Type 1 ligation. Macrophage colony stimulating factor, the primary macrophage survival and differentiation factor, does not activate NF-κB or protect macrophages from MTX-induced apoptosis. A weak NF-κB activator, Receptor Activator of NF-κB Ligand (RANKL) is likewise ineffective. Blocking NF-κB in TNF-α-exposed macrophages allowed pro-apoptotic actions of TNF-α to dominate, even in the absence of MTX. MTX itself does not promote apoptosis through interference with NF-κB signaling.

**Conclusions:**

These findings provide another mechanism by which TNF-α sustains macrophage numbers in inflamed tissue and identify a further point of clinical complementarity between MTX and anti-TNF-α treatments for rheumatoid arthritis.

## Introduction

Synovial inflammatory macrophages have a central role in maintaining disease activity in rheumatoid arthritis (RA). Macrophage numbers in tissue are regulated by recruitment, local proliferation, local cell death and emigration to draining lymph nodes [1,2]. Synovial macrophage apoptosis has also been observed in synovium in RA [3,4]. A suppressed rate of apoptosis would contribute to maintaining inflammatory macrophage numbers, and thus clinical activity, in macrophage-dependent conditions. An enhanced rate of synovial macrophage apoptosis is reported in RA patients responding to anti-TNF-α treatments [4] as a delayed, rather than an early phenomenon [5]. Macrophage apoptosis has also been reported in patients with Crohn's disease after anti-TNF-α treatment [6]. These observations suggest that TNF-α directly, or indirectly, sustains macrophage survival in these conditions.

TNF-α activity supports recruitment of macrophages into RA synovium [7], but is not known to enhance the proliferation of macrophages or to prevent the emigration of macrophages through lymphatics [2]. TNF-α, however, may deliver either apoptotic or survival signals, depending on the cell context. TNF-α is a ligand for two related receptors, TNF-R1 (p55) and TNF-R2 (p75). Macrophages and their bone marrow and blood-borne progenitors express both receptors [8]. TNF-α ligation to TNF-R1 leads to assembly of a Death-Inducing Signaling Complex (DISC) and ultimately activation of downstream effector caspases-3/6/7. Where this is the dominant consequence of TNF-R1 ligation, apoptosis follows. In other circumstances, TNF-R1 initiates survival signaling [9] through activation of the NF-κB pathway. TNF-α ligation to TNF-R1 can thus bring apoptosis or survival, depending on the relative activities generated in the DISC-initiated and NF-κB pathways [10]. Macrophage survival is notably dependent on NF-κB signaling. The transcriptionally active RelA/NF-κB_1 _(p65/p50) complex appears in macrophage nuclei constitutively during late differentiation and is important for continuing survival [10,11]. *In vivo*, there is enhanced nuclear expression of RelA/NF-κB_1 _in synovial macrophages in RA [12], consistent with a role for NF-κB activity in maintaining macrophage survival.

The observations of enhanced apoptosis during anti-TNF-α treatment imply that TNF-α predominantly antagonises apoptosis in RA and Crohn's disease [4,6]. On the other hand, methotrexate (MTX), an anti-rheumatic drug that has demonstrable pro-apoptotic effects on monocyte/macrophage cells *in vitro*, does not appreciably alter the synovial membrane macrophage apoptosis rate in RA patients [3,13]. This led us to question whether the presence of TNF-α in synovium was providing a survival signal to macrophages in MTX-treated patients, thus antagonizing one potential therapeutic function of MTX. That would provide additional explanation for the clinical complementarity of MTX and anti-TNF-α treatments in RA [14]. In the study of Catrina *et al.*, which demonstrated synovial macrophage apoptosis in response to anti-TNF-α therapy, the majority of patients were co-treated with MTX [4].

MTX is a folate analogue with numerous effects on inflammatory cell proliferation and survival, cytokine expression, angiogenesis, cell adhesion and reactive oxygen production [15]. The known direct targets of MTX are dihydrofolate reductase, thymidylate synthase and 5-aminoimidazole-4-carboxamide ribonucleotide (AICAR) transformylase [15]. Consequences of inhibiting these enzymes include reduced availability of purines and pyrimidines for DNA and RNA synthesis and accumulation of AICAR. AICAR, by inhibiting enzymatic deamination of adenosine and adenosine monophosphate, is proposed to increase availability of adenosine extracellularly, for anti-inflammatory effect through adenosine cell surface receptors [16]. These effects are generally believed to be mediated by long-lived polyglutamate forms of MTX, which are generated in many cell types, including normal myeloid precursor cells and myelocytic cancer cells [17].

## Materials and methods

### Materials

The Recombinant Macrophage-Colony Stimulating Factor (M-CSF) was obtained from R&D Systems (Sydney, NSW, Australia). Annexin V conjugated to phycoerythrin (Annexin V-PE) and 7-amino-actinomycin (7-AAD) were from BD Biosciences (Sydney, NSW, Australia). Parthenolide was purchased from Alexis Biochemicals (Lausen, Switzerland). Rabbit anti-IκBα (C-21) polyclonal antibody, anti-NF-κB p65, anti-NF-κB p50, anti-c-Rel and anti-RelB were obtained from Santa Cruz Biotechnology, Santa Cruz, CA, USA. Mouse anti-phospho-IκBα (Ser32/36) (5A5) and horseradish peroxidase-conjugated anti-rabbit were from Amersham Biosciences (Sydney, NSW, Australia). Methotrexate was provided by Mayne Pharma Pty Ltd. (Adelaide, South Australia, Australia). Etanercept came from Wyeth Australia Pty Ltd. (Sydney, NSW, Australia). Recombinant GST-rRANKL was kindly provided by Ming-Hao Zheng and Jiake Xu, Centre for Orthopaedic Research, University of Western Australia [18]. All other reagents were sourced from Sigma-Aldrich (Sydney, NSW, Australia), unless otherwise indicated.

### Cell culture

RAW_264.7 _cells were obtained from the American Type Culture Collection and maintained in Dulbecco's modified Eagle's medium (Life Technologies, Carlsbad, California, USA) with 10% low endotoxin fetal calf serum (CSL, Melbourne, Australia), penicillin and gentamicin (DMEM-FCS). Bone marrow-derived monocytes/macrophages (BMDM) were collected from the femora and tibiae of eight-week old female C57BL or BALB/c mice, as previously described [19], with the approval of the Animal Ethics Committee of The University of Western Australia. Cells were expanded in M-CSF (30 ng/mL)-supplemented DMEM-FCS for seven days at 37°C/5% CO_2_/95% air. The experiments reported here were conducted with BMDM from C57BL mice, but results were comparable using BALB/c mice.

### MTT cytotoxicity assay

Viability was estimated using the 3-(4,5-dimethyl-2-thiazolyl)-2,5-diphenyl-2H-tetrazoliumbromide (MTT) assay. BMDM (3 × 10^4 ^cells/well) were seeded in 24-well plates in M-CSF-supplemented DMEM-FCS. After 48 hr, cells were treated with TNF-α for 3 hr, followed by MTX exposure for 24 hr. At the end of treatments, 50 μL of MTT (5 mg/mL) was added to each well, and cells were incubated at 37°C for 2 hr. Formazan crystals were then solubilised with 150 μL of 44% dimethyl formamide/20% SDS at room temperature for at least 30 minutes on a rocking platform. A total of 100 μL aliquots were quantitated at 550 nm using a microplate reader (POLARstar OPTIMA, BMG, Germany). Triplicate assays were conducted for all conditions.

### Caspase-3 protease activity

RAW_264.7 _cells and BMDM were cultured in six-well plates at a density of 0.3 × 10^6 ^cells/well for 48 hr. After treatments, trypsin-detached RAW_264.7 _cells and scraped BMDM were lysed and assayed for caspase-3 activity as described previously [20].

### Flow cytometry analysis

Apoptotic cells were quantitated by staining with annexin V-PE and 7-AAD, as specified by the manufacturer (BD Biosciences). Flow cytometry was performed on populations of 5000-10,000 cells (Becton Dickinson FACSCalibur, Sydney, NSW, Australia), with fluorescence of annexin V-PE and 7-AAD measured with a 585/42 nm bandpass filter (FL2 channel) and a 670 nm longpass filter (FL3 channel), respectively. Data are expressed as percentages of apoptotic cells, as defined by annexin-V-PE positivity and 7-AAD negativity.

### Cell cycle analysis

RAW_264.7 _cells and BMDM (0.25 × 10^6^/well of a 12-well plate) were collected and washed in cold Dulbecco's Phosphate Buffered Saline (DPBS) before being fixed with 70% ethanol at -20°C. After 24 hr, cells were centrifuged at 500 × *g *for 10 minutes at 4°C and resuspended in 500 μL of staining buffer (50 μg/mL propidium iodide and 25 μg/mL RNase in 0.1% Triton X-100). After incubating at 37°C for 30 minutes, cells were analysed on a FACSCalibur flow cytometer (Becton Dickinson) using a doublet discrimination protocol.

### Western blot analysis

RAW_264.7 _cells and BMDM (1 × 10^6 ^per treatment) were washed with ice-cold DPBS and lysed in RIPA buffer (50 mM Tris, pH 7.5, containing 150 mM NaCl, 1% IGEPAL, 1% sodium deoxycholate, 0.1% SDS and 10 mM EDTA) supplemented with 1X complete protease inhibitors (Roche Applied Science, Sydney, NSW, Australia). A total of 20 μg of total protein was separated by SDS-PAGE and transferred to Hybond-P PVDF membranes (Amersham Biosciences). Membranes were blocked in 1% BSA/5% non-fat milk in TTBS (10 mM Tris, pH 7.6, 150 mM NaCl, 0.1% Tween 20) for one hour at room temperature, before being probed with specific antibodies to phospho-IκBα (1:1000 dilution), IκBα (1:2,000) or β-actin (1:8,000). Horseradish peroxidase-conjugated anti-rabbit and anti-mouse were used at 1:8,000 and 1:20,000 dilutions, respectively. All antibodies were prepared in SignalBoost Immunoreaction Enhancer (Calbiochem, Darmstadt, Germany) and applied for one hour at room temperature. Blots were revealed with enhanced chemiluminescence (ECL) reagents (Amersham Biosciences).

### Electrophoretic Mobility Shift Assay (EMSA)

EMSA was performed on nuclear extracts from RAW_264.7 _cells (8 × 10^6 ^per treatment) that were treated with 10 ng/mL TNF-α for up to one hour, according to previously described protocols [21]. A double-stranded NF-κB consensus oligonucleotide probe 5'-GGGCATGGGAATTTCCAACTC-3' (0.25 pmol) with 5'-G overhangs was filled in with labeled (α-^32^P)dCTP (Amersham Pharmacia Biotech, Buckinghamshire, England) using the Klenow fragment of *E. coli *DNA polymerase I (Promega, Madison, Wisconsin, USA). This DNA probe was incubated with 3 μg of nuclear proteins for 10 minutes at room temperature. Where indicated, antibodies (1 μg) to specific NF-κB factors or unlabelled oligonucleotide probes at 100-fold molar excess were also included for supershift EMSA and competition experiments, respectively. Samples were loaded onto a 4% polyacrylamide gel, containing 0.25X Tris-Borate-EDTA buffer, which had been pre-run for two hours in the same buffer. After separation, gels were exposed to Cronex X-ray film, using a single intensifying screen.

### Transient transfection of RAW_264.7 _cells and BMDM with an NF-κB reporter plasmid

RAW_264.7 _cells (1 × 10^7^) were transiently transfected with 0.5 μg of pNFκB-TA-Luc (Mercury™ Pathway profiling system, BD Biosciences) by the DEAE-dextran procedure, as previously described [21]. Transfected cells were resuspended in DMEM-FCS and distributed into a 24-well culture plate at a density of 3.3 × 10^5 ^cells/well. Cells were rested for 48 hr before experimentation. Firefly luciferase expression in transfected RAW_264.7 _cells was measured using the Promega Luciferase Assay System, according to the manufacturer's instructions. Promoter activity was quantified by measuring light production in a multifunctional microplate reader (POLARstar OPTIMA, BMG, Germany), and presented as relative light units (RLU).

BMDM were transfected with Lipofectamine™ and Plus reagents (Invitrogen) according to manufacturer's instructions. Briefly, for each 24-well, 1 μg pNF-κB-TA-Luc was combined with 0.5 μL Plus reagent and 2 μL Lipofectamine™ in antibiotic-free medium. A total of 1.5 × 10^5 ^cells/well was added to the transfection mixture and plated in M-CSF-containing DMEM-FCS. After four to six hours of incubation at 37°C, wells were replaced with fresh growth medium. Luciferase expression was measured at least 48 hr after transfection.

### Statistical analysis

Statistical significance (*P *< 0.05) between groups of experimental data was assessed using paired Student's *t*-tests.

## Results

### TNF-α protects primary macrophages and a macrophage cell line from MTX-induced apoptosis

**Figure 1:**
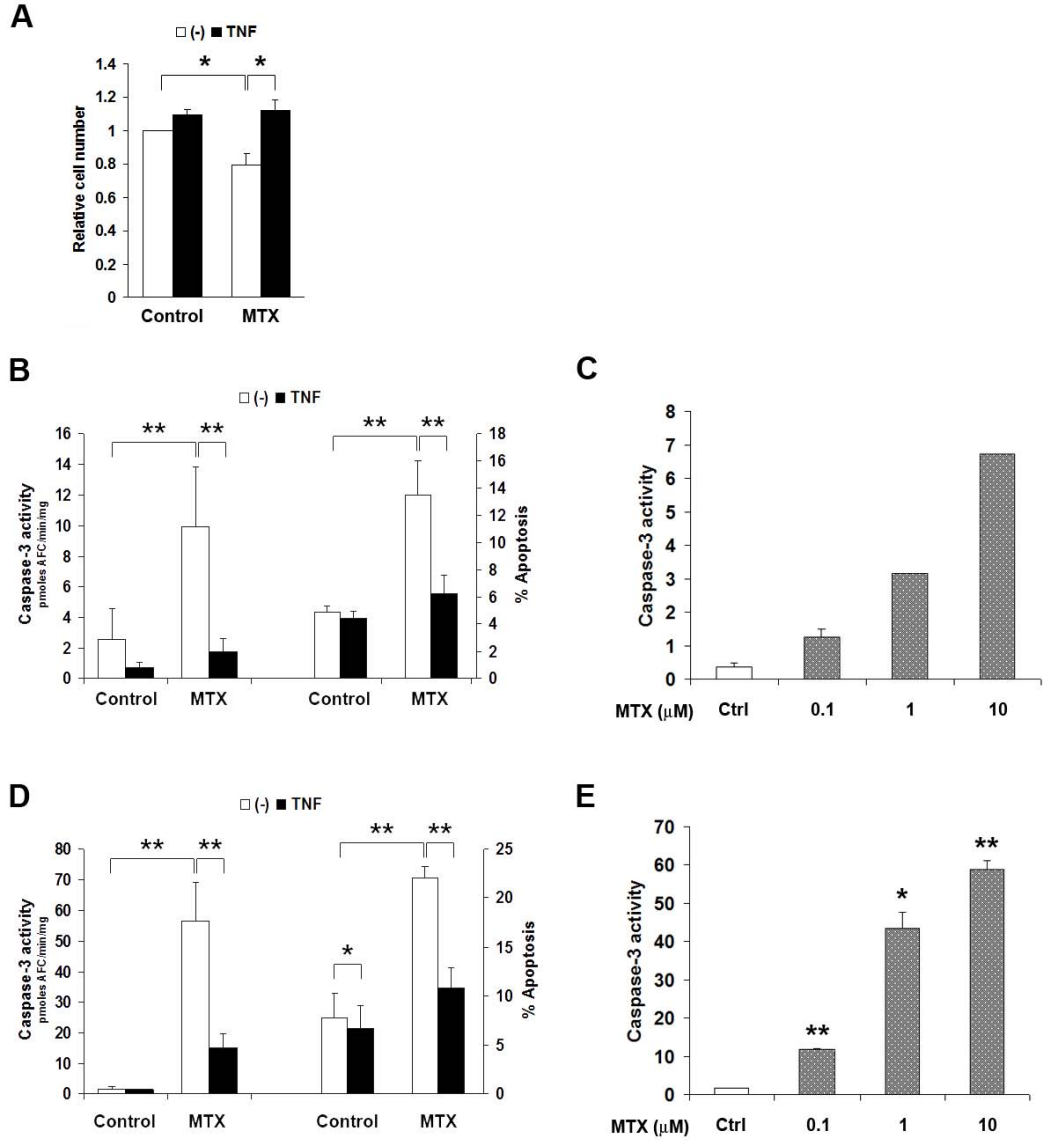
**TNF-a antagonises MTX-induced apoptosis in macrophages, independent of M-CSF action**. **A**. Exposure of primary BMDM to 10 mM MTX for 24 hr in the presence of 30 ng/mL M-CSF resulted in loss of approximately 25% of cells. This could be entirely prevented by introducing TNF-a three hours before MTX. Cell viability was assessed by the MTT reduction assay. Shown are mean ± SEM of normalised data from three independent experiments. **B **and **D**, TNF-a suppresses caspase-3 activation (left axis) and annexin-V binding (right axis) in MTX-exposed primary BMDM (B) and RAW_264.7 _cells (D). MTX was added three hours after TNF-a treatment, and caspase-3 activity or annexin-V binding was measured after 24 hr for BMDM and 6 hr for RAW_264.7 _cells. Data are mean ± SEM of at least four independent experiments in each case. **C **and **E**, MTX dose-dependently increases apoptosis in BMDM (**C**) and RAW_264.7 _cells (**E**). * = *P *< 0.05; ** = *P *< 0.01.

M-CSF is the primary growth, differentiation and survival factor for macrophages under physiological conditions. Serum concentrations of M-CSF are elevated to approximately 0.6 ng/ml in patients with RA [22], but concentrations in synovium are unknown. *In vitro*, a concentration of 30 ng/ml was optimal for sustaining the growth and survival of bone marrow-derived macrophages (BMDM) of C57BL/6 mice (results not shown). In the presence of 30 ng/mL M-CSF, exposure to 10 μM MTX for 24 hr caused an approximately 25% loss of cell numbers in BMDM cultures (Figure 1A). This was related to increased apoptosis, as estimated by caspase-3 activity (Figure 1B), annexin-V binding (early apoptosis; Figure 1B) and increased sub-G_0 _(apoptotic) fraction on flow cytometric cell cycle analysis (Table 1). The proportion of cells in S-phase also increased with MTX, consistent with its known action on completing DNA synthesis [23] (Table 1).

**Table 1:** Cell cycle analysis of BMDM treated with TNF-α or its control, followed by MTX exposure for 24 hr or M-CSF withdrawal for 48 hr*.

	*Cell cycle distribution (% of cells)*			
	*sub G0*	*G0/G1*	*S*	*G2/M*
Control	3.5 ± 0.5	78.6 ± 2.9	6.0 ± 1.0	10.7 ± 2.7
MTX (10 μM)	7.5 ± 1.0^a^	62.7 ± 2.2^a^	16.0 ± 1.2^a^	12.0 ± 1.2
TNF-α (10 ng/mL)	3.6 ± 0.4	77.0 ± 2.2	7.5 ± 1.7	10.7 ± 2.0
TNF-α + MTX	5.8 ± 0.8^b^	65.7 ± 4.8	13.8 ± 3.2	13.1 ± 2.6
				
M-CSF (30 ng/mL)	1.5 ± 0.4	74.2 ± 2.6	8.4 ± 1.9	13.9 ± 2.2
- M-CSF	15.4 ± 4.1^a^	74.7 ± 5.1	2.8 ± 0.7^a^	5.5 ± 0.9^a^
M-CSF + TNF-α	1.5 ± 0.2	71.8 ± 1.9	9.3 ± 1.3	15.3 ± 2.7
- M-CSF + TNF-α	4.4 ± 2.3^b^	82.3 ± 3.7^b^	1.9 ± 0.4	9.7 ± 2.7

* Results are mean ± SEM of at least three independent experiments in each case. ^a ^*P *< 0.05 compared to controls. ^b ^*P *< 0.05 compared to MTX alone or no M-CSF.

BMDM, bone marrow-derived macrophages; M-CSF, macrophage colony stimulating factor; MTX, methotrexate; TNF-α, tumor necrosis factor-alpha.

Caspase-3 activation was evident at concentrations as low as 0.1 μM MTX in BMDM, with activity increasing dose-dependently up to 10 μM (Figure 1C). MTX, therefore, induces macrophage apoptosis at levels usually attained in treated human RA patients [24]. This was replicated in the murine RAW_264.7 _macrophage cell line (Figure 1E). RAW_264.7 _macrophages do not require exogenous M-CSF to proliferate, having gained growth signaling through introduction of the Abelson leukaemia virus [25]. They have been previously found to model primary macrophage apoptosis response [26]. A concentration of 10 μM MTX was used in subsequent experiments.

TNF-α could also maintain the viability of macrophages exposed to 10 μM MTX. TNF-α, when introduced three hours before MTX, completely prevented the loss of cell numbers in primary BMDM cultures exposed to MTX (Figure 1A). TNF-α also almost completely inhibited MTX-induced caspase-3 activation (Figure 1B) and annexin-V binding (Figure 1B) and significantly suppressed the sub-G_0 _(apoptotic) fraction on flow cytometric cell cycle analysis of MTX-exposed BMDM (Table 1). TNF-α did not reverse the MTX-induced increase in cells in S-phase. TNF-α, therefore, protected from MTX-induced cell loss by countering apoptosis, not through any action on cell proliferation. TNF-α also reduced the sub-G_0 _population of M-CSF-deprived BMDM, without restoring S phase progression (Table 1). It is notable that TNF-α was protective even in the presence of M-CSF concentrations that are optimal for apoptosis prevention, suggesting distinct pathways for apoptosis protection. Further increases in M-CSF concentration in culture, up to 120 ng/mL, failed to substitute for the anti-apoptotic action of TNF-α (results not shown). This suggested an action that was independent of M-CSF intracellular signaling and removed the possibility that TNF-α was acting through autocrine induction of M-CSF secretion. At least three hours of pre-exposure to TNF-α was required for optimal protection from MTX-induced apoptosis (results not shown).

TNF-α provided comparable protection from MTX-induced apoptosis in the RAW_264.7 _macrophage cell line. TNF-α exposure significantly (*P *< 0.05) suppressed spontaneous apoptosis of RAW_264.7 _cells as measured by caspase-3 activity and annexin-V binding (Figure 1D). RAW_264.7 _cells shared the susceptibility of primary macrophages to MTX, demonstrating enhanced caspase-3 activity and annexin-V binding after six hours of exposure (Figure 1D). This increase was markedly suppressed when TNF-α was added three hours before MTX (Figure 1D). Thus, the anti-apoptotic effect of TNF-α is replicated in RAW_264.7 _cells, allowing use of this cell line for studying the phenomenon.

### TNF-α protection from apoptosis is mediated through TNF-R1 (p55), not TNF-R2 (p75) receptor activation

**Figure 2:**
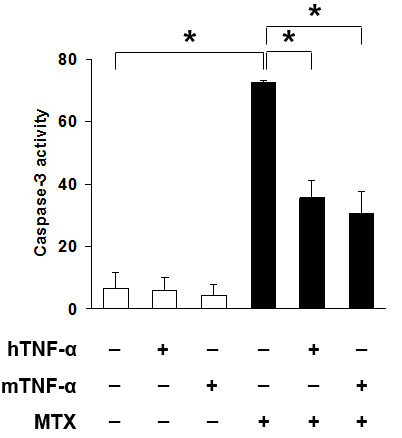
**Human (h) and murine (m) TNF-a confer comparable protection against apoptosis in RAW_264.7 _cells exposed to 10 mM MTX**. Caspase-3 activity was measured after six hours with MTX, without TNF-a, or pre-treated with 10 ng/mL mTNF-a or hTNF-a for three hours. Shown are mean ± SEM of three independent experiments. * = *P *< 0.05.

Monocytes/macrophages express both TNF-R1 and TNF-R2 [8]. To determine the receptor subtype(s) that mediated the survival effect of TNF-α, we exploited the species specificity of TNF-α action. Murine TNF-α (mTNF-α) activates both TNF-R1 and TNF-R2 on murine macrophages, while human TNF-α (hTNF-α) activates TNF-R1 only, thereby providing a means to distinguish effects that are mediated through different receptors [27]. mTNF-α and hTNF-α provided comparable protection from MTX-induced caspase-3 activation (Figure 2, *P *> 0.05), indicating that protection is mediated through TNF-R1, and does not require TNF-R2 signaling.

### NF-κB is required for the survival effect of TNF-α in macrophages

TNF-R1 ligation activates several pro-survival pathways via its association with TRAF2 (TNFR-Associated Factor 2), RIP (Receptor-Interacting Protein) c-Src and Jak2. These include the NF-κB pathway, Phosphatidylinositol-3-Kinase/Protein Kinase B (PI3K/AKT) and Mitogen-Activated Protein Kinase pathways [28,29]. Constitutive NF-κB activity is critical to macrophage survival [26]. Therefore, we next examined the kinetics of NF-κB activation in TNF-α-stimulated macrophages and investigated whether NF-κB activity was required for the anti-apoptotic effect of TNF-α.

**Figure 3:**
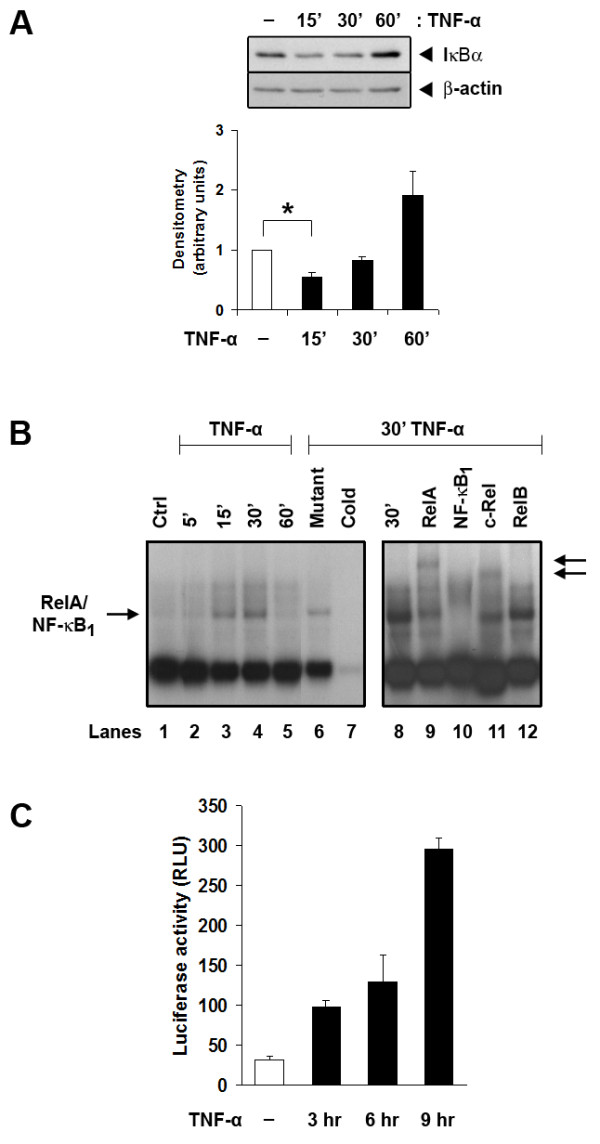
**TNF-a activates NF-kB in RAW_264.7 _cells**. **A**. TNF-a promotes degradation of IkBa and resynthesis over 60 minutes. IκBα and β-actin protein levels were assessed by Western blot analysis and quantified by densitometry (lower panel). Densitometry data show IκBα levels that were corrected by β-actin and represent normalised data from four independent experiments. * = *P *< 0.05. **B**. EMSA for NF-kB proteins in nuclear extracts of RAW_264.7 _cells over six hours of TNF-a exposure. The arrow on the left indicates the position of the RelA/NF-kB_1 _heterodimer. Competition for binding by an unlabeled specific oligonucleotide (lane 7) but not by an irrelevant sequence at 100-fold molar excess (lane 6) confirmed specificity of DNA binding. Supershift analyses with specific antibodies to RelA, NF-kB_1 _(p50), c-Rel and RelB (lanes 9, 10, 11 and 12, respectively, of the right panel) confirmed the identities of RelA and NF-kB_1 _in the complex with a lighter supershift c-Rel band. Arrows on the right indicate the locations of supershifted bands. The figure is representative of two independent experiments. **C**. TNF-a (10 ng/ml) induces NF-kB activity, as estimated by luciferase activity in RAW_264.7 _cells that had been transiently transfected with the pNF-kB-TA-Luc reporter. Results are representative of three independent experiments.

Proteosomal degradation of the NF-κB inhibitory protein, IκBα, is an early indicator of activation in the canonical NF-κB pathway [30]. TNF-α (10 ng/mL) addition in RAW_264.7 _cells induced the degradation of IκBα within 15 minutes (Figure 3A, *P *< 0.05). This corresponded to the appearance of DNA-binding NF-κB in the nucleus, with activity peaking at 30 minutes before returning to baseline levels by one hour (Figure 3B). Supershift analyses with specific antibodies to RelA, NF-κB_1_, c-Rel and Rel-B identified RelA (p65) and NF-κB_1 _(p50) as the main components of the NF-κB complex induced by TNF-α (Figure 3B). A light c-Rel supershifted band was also detected (Figure 3B). IκBα levels recovered by one hour (Figure 3A). The *IKBA *gene is also NF-κB-responsive, so early recovery of IκBα levels indicates functional NF-κB signaling [30]. Lastly, TNF-α stimulation of RAW_264.7 _cells transiently expressing a NF-κB-responsive reporter construct showed that NF-κB transcriptional activity was enhanced in a time-dependent manner (Figure 3C). These results confirm that TNF-α is a rapid activator of NF-κB function in macrophages.

**Figure 4:**
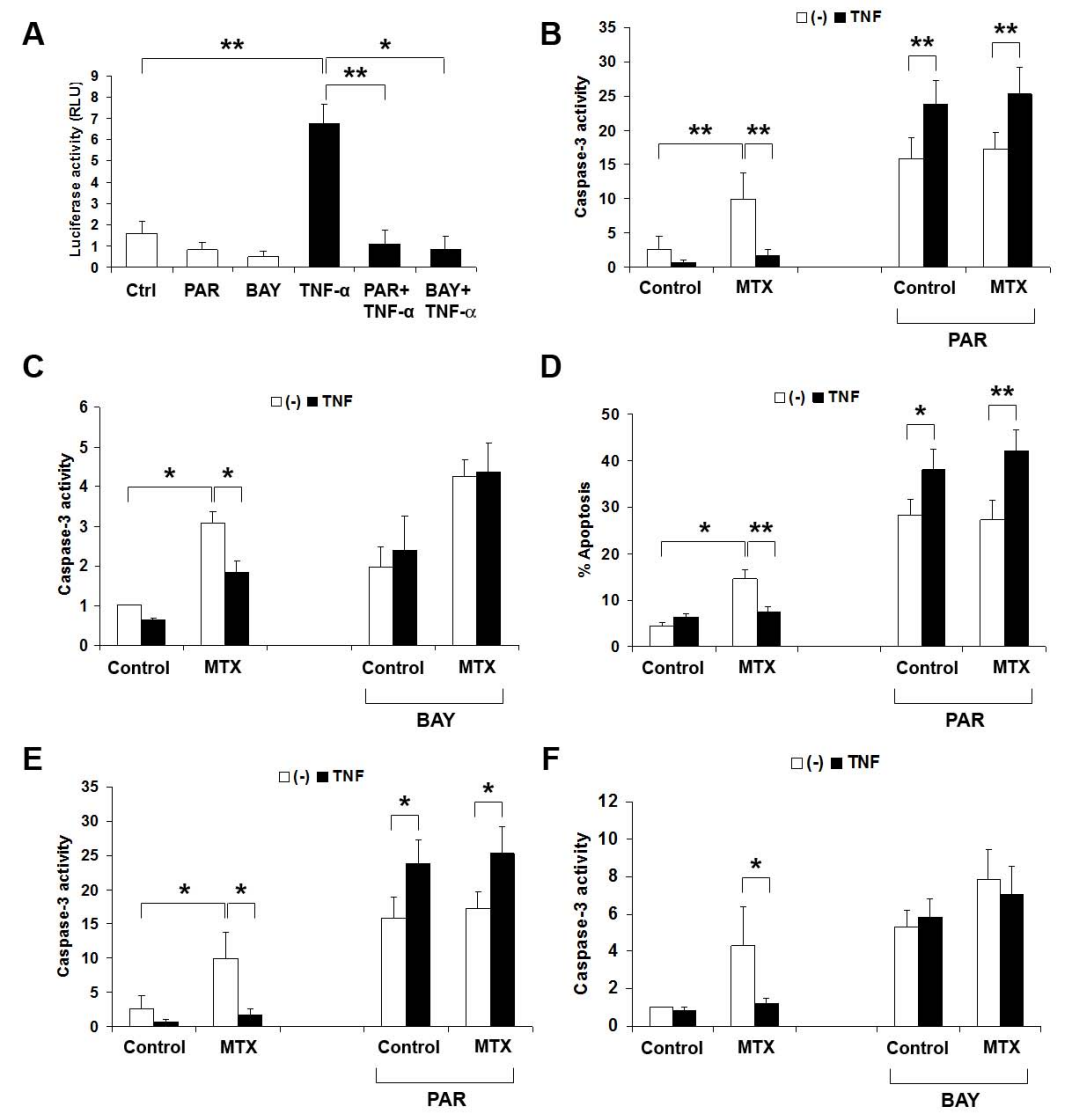
**TNF-a must activate NF-kB signaling to protect macrophages from MTX-induced apoptosis**. **A**. PAR and BAY (10 μM) prevent TNF-a-induced NF-kB activity in RAW_264.7 _cells, as measured by activity of the pNF-kB-TA-Luc reporter. Inhibitors were introduced 30 minutes before TNF-a and luciferase activity was measured nine hours later. Results are mean ± SEM of at least four independent experiments. **B-F**. PAR and BAY (10 mM or 7.5 mM) prevent TNF-a from rescuing MTX-treated RAW_264.7 _cells (B and C) and BMDM (D-F) from apoptosis. Cells were exposed to PAR (B, D, and E) or BAY (C and F) for 30 minutes before TNF-a stimulation for three hours, after which MTX was introduced. Annexin-V binding and/or caspase-3 activity was measured 6 hours later in RAW_264.7 _cells and 24 hours later in BMDM. Results are mean ± SEM of at least three independent experiments in each case. Normalised data are shown for BAY experiments. * = *P *< 0.05; ** = *P *< 0.01.

To investigate whether NF-κB activity is essential for TNF-α activity against MTX-induced apoptosis, the sesquiterpene lactone parthenolide (PAR) and BAY11-7085 (BAY) were used to specifically prevent NF-κB signaling [19,31]. PAR and BAY pre-treatment for 30 minutes abolished both constitutive and TNF-α-stimulated NF-κB transcriptional activity (Figure 4A). They also completely prevented TNF-α from rescuing RAW_264.7 _cells (Figure 4B,C) and primary macrophages (Figure 4D-F) from MTX-induced apoptosis and caspase-3 activation. These findings indicate that NF-κB activation is required for TNF-α protection against apoptosis induced by MTX. Notably, TNF-α became pro-apoptotic, rather than anti-apoptotic when NF-κB signaling was blocked, when estimated by either annexin-V binding (*P *< 0.05) or caspase-3 activity (*P *< 0.05).

The involvement of PI3K/AKT, ERK, JNK and p38 MAP kinases was also explored using their specific inhibitors, but they were found to be dispensable for TNF-α-induced survival of macrophages, as assessed by annexin-V staining and caspase-3 activity (results not shown).

### Autocrine TNF-α signaling is not required for basal survival of M-CSF-maintained macrophages

Macrophages are also sources of TNF-α, raising the possibility that constitutive NF-κB activity (and thus survival) depended on autocrine TNF-α stimulation. We, therefore, treated BMDM cultures with etanercept, a chimeric protein comprising Fc domains of human IgG_1 _and TNF-α-binding domains of the Type 2 TNF receptor, which neutralises both murine and human TNF-α [32]. An irrelevant human myeloma-derived IgG_1 _served as a control. Etanercept, however, did not increase caspase-3 activation, indicating that autocrine TNF-α stimulation was not important for survival of macrophages cultured with optimal concentrations of M-CSF (results not shown).

### RANKL, a weak activator of NF-κB in macrophages, does not protect from MTX-induced apoptosis

**Figure 5:**
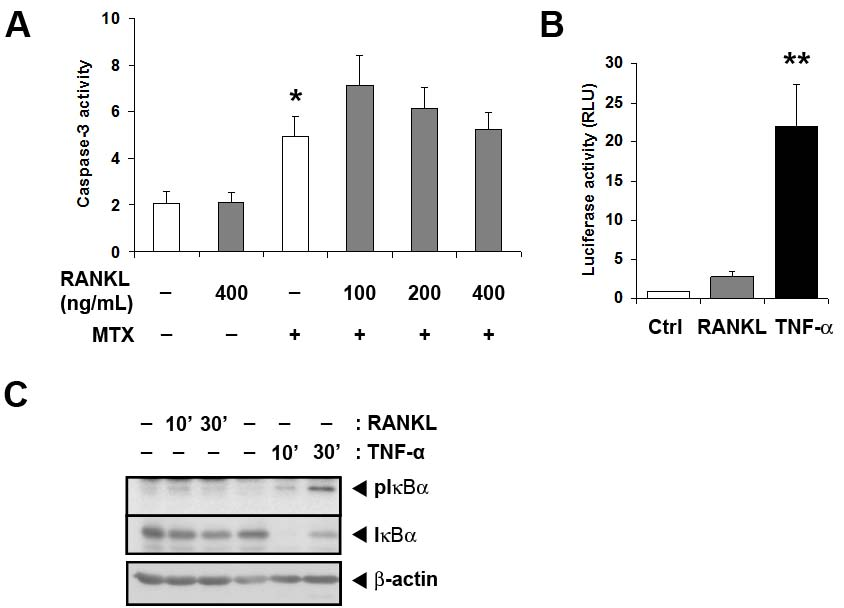
**RANKL, a weak NF-κB activator, does not protect primary BMDM from MTX-induced apoptosis**. **A**. RANKL (100 to 400 ng/mL) was introduced to BMDM 6 hours before MTX (10 mM) treatment. Caspase-3 activity was measured 24 hours later. Results are mean ± SEM from at least six independent experiments. **B**. and **C**. Compared to RANKL (200 ng/mL), TNF-a (10 ng/mL) treatment causes greater activation of NF-kB in BMDM. Cells transiently expressing the pNF-kB-TA-Luc reporter were stimulated with RANKL or TNF-a for six hours (B). Shown are mean ± SEM of normalised data from three independent experiments. C. Cells were M-CSF-starved overnight before stimulation with RANKL or TNF-a for 10 minutes and 30 minutes. Whole-cell lysates were subjected to western blot analysis for phosphorylated IkBa, total IkBa and b-actin. Results are representative of three independent experiments. * = *P *< 0.05; ** = *P *< 0.01.

The observation that TNF-α protected macrophages from MTX-induced apoptosis led us to question whether other cytokines present in rheumatoid synovium or erosions may also protect through NF-κB induction. Receptor Activator of NF-κB Ligand (RANKL) circulates at elevated concentration in RA and is demonstrable in rheumatoid synovium and erosions [33]. It is required for osteoclast formation from monocyte/macrophage precursors. Unlike TNF-α, however, RANKL did not suppress caspase-3 activation in MTX-exposed primary macrophage cultures at concentrations up to 400 ng/mL (Figure 5A). A dose of 200 ng/mL of RANKL is sufficient to elicit classical osteoclastogenesis in our hands [34], so was used for other experiments. Comparing effects on NF-κB activation, we found that RANKL (six hours) stimulated an approximately three-fold increase in NF-κB-luciferase reporter expression in transfected BMDM, well short of the approximately 22-fold increase with TNF-α (Figure 5B). Western blot analyses also indicated weaker phosphorylation and degradation of IκBα after RANKL treatment, compared to TNF-α (Figure 5C). Therefore, RANKL does not protect differentiated primary macrophages from MTX, possibly due to insufficient activation of NF-κB.

### MTX itself does not induce apoptosis through NF-κB suppression

**Figure 6:**
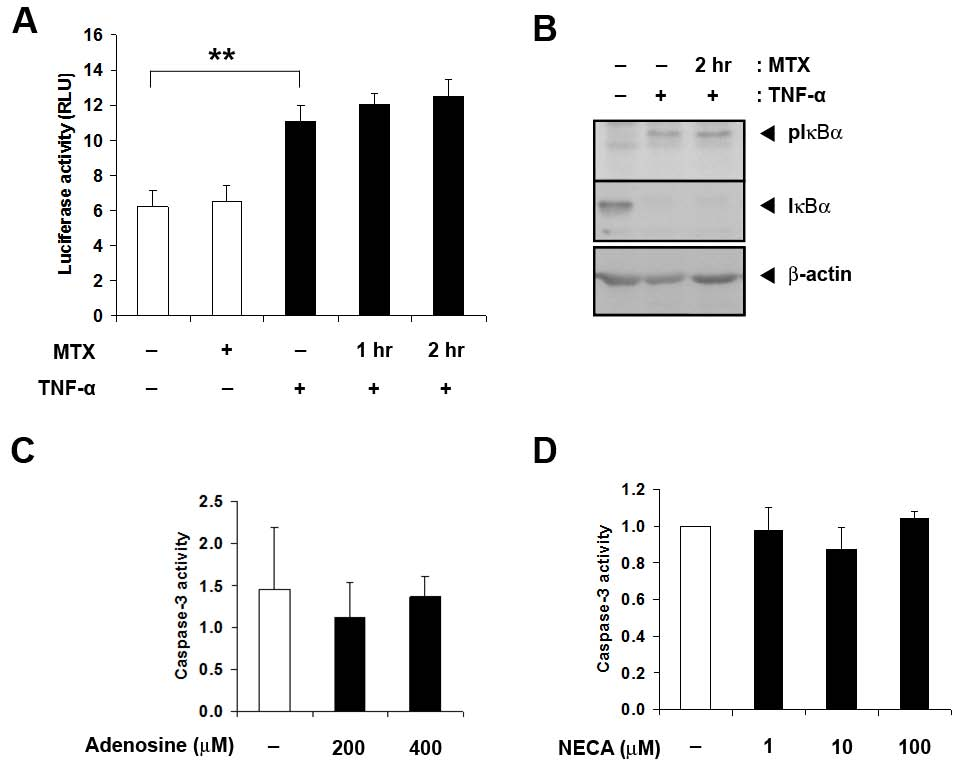
**MTX does not inhibit TNF-a-induced NF-kB activity**. **A**. Reporter-expressing RAW_264.7 _cells were exposed to MTX for one hour or two hour before TNF-a stimulation for a further three hour. Shown are mean ± SEM of four independent experiments. **B**. BMDM were pre-treated with MTX for two hour before TNF-a stimulation for 10 minutes. Whole-cell lysates were probed with antibodies for phosphorylated IkBa, total IkBa and b-actin. **C**. Adenosine and **D**. NECA do not increase basal macrophage apoptosis. BMDM were exposed to increasing concentrations of adenosine or NECA for 24 hour before caspase-3 measurements. Shown are mean ± SEM of results from three independent experiments in each case. ** = *P *< 0.01.

The observation that NF-κB induction countered the apoptotic action of MTX led us to test whether MTX itself acted through inhibiting NF-κB. This has been previously observed in Jurkat T-cells and in the poorly differentiated myelo-monocytic U937 cell line [35]. However, MTX, at concentrations sufficient to induce apoptosis, failed to suppress either basal or TNF-α-induced NF-κB activity in RAW_264.7 _cells transiently transfected with a specific NF-κB reporter construct (pNF-κB-TA-Luc) (Figure 6A). In BMDM, it also failed to prevent the TNF-α-stimulated phosphorylation of IκBα, an essential step in IκBα degradation and RelA/NF-κB_1 _complex release (Figure 6B). MTX, therefore, does not exert its apoptotic actions through NF-κB suppression.

Anti-inflammatory actions of MTX on macrophages have been attributed to enhanced extracellular adenosine generation. However, neither adenosine itself (Figure 6C), or the pan-adenosine receptor agonist NECA (Figure 6D), affected caspase-3 activity. Thus, adenosine accumulation alone is not a sufficient explanation for MTX-induced macrophage apoptosis.

## Discussion

TNF-α protects macrophages from apoptosis induced by MTX. This offers an explanation for the clinical observation that anti-TNF-α treatments cause apoptosis of monocyte/macrophage lineage cells in peripheral blood and synovium of patients with RA [4], in a study where most participants were also taking MTX. The survival signal from TNF-α is transduced through TNF-R1 and the canonical NF-κB pathway. M-CSF, which directs macrophage differentiation, growth and survival, but does not activate NF-κB signaling, cannot substitute for TNF-α. This is notable, because M-CSF is present in peripheral blood and synovial fluid in RA [22], and might have been expected to render anti-apoptotic effects of TNF-α redundant. M-CSF signals for growth and survival primarily through the PI3K/AKT pathway.

TNF-α can activate both pro-apoptotic and anti-apoptotic pathways, with outcomes that differ between cell types and conditions. The importance of NF-κB signaling in survival is highlighted by the outcomes of PAR and BAY exposure, which converted TNF-α from a dominantly pro-survival signal to an apoptotic signal. NF-κB-regulated proteins that have been linked to inhibition of apoptotic signaling include TRAF-1, TRAF-2, cIAP-1, cIAP-2, XIAP, FLIP, A20, GADD45β, the antioxidant Mn-SOD, and anti-apoptotic members of the Bcl-2 family, such as A1 and Bcl-xL [36].

The canonical NF-κB pathway is activated in macrophages of rheumatoid synovium [12], consistent with a role in maintaining cell survival in untreated patients [10,11]. Other products of the inflamed synovium, including IL-1, RANKL, GM-CSF, VEGF, PDGF and leukotrienes [37] can promote NF-κB signaling in responsive cells and may contribute, with TNF-α, to NF-κB activation in macrophages of rheumatoid synovium. However, the experiments with RANKL indicated that not all NF-κB activators protect from apoptosis. RANKL failed to protect primary murine macrophages from MTX-induced apoptosis, even at concentrations that promote osteoclastogenesis in our hands [34] and which exceed the concentrations reported in rheumatoid synovial fluid [38]. The difference may arise because RANKL activates NF-κB less intensely, or because of differences in other pathways recruited by TNF-R1 and RANK. Finding that RANKL is not a survival factor for macrophages mirrors findings with osteoclast precursors, where RANKL promotes activation and differentiation, but not survival [39]. The mature osteoclast, however, is protected from apoptosis by RANKL with mixed reports of NF-κB involvement [40]. The finding that RANKL-induced NF-κB activation is insufficient to protect primary macrophages from apoptosis is also in accord with the conclusion from clinical studies, that RANKL is not an important driver of synovial inflammation in RA, even though it is critical for erosion formation [41].

MTX has been reported to suppress NF-κB activity in a different cell context [35]. However, NF-κB suppression could not be demonstrated in MTX-exposed macrophages, so cannot be the mechanism of MTX-induced macrophage apoptosis. MTX anti-inflammatory response has also been linked with the extracellular accumulation of adenosine. Adenosine is reported to promote apoptosis of colon cancer cells *in vitro *[42], but we could find no evidence that adenosine alone causes apoptosis in macrophages. The experiments could not disprove a permissive role for adenosine. The experiments do, however, prove that MTX and TNF-α do not just act conversely on NF-κB signaling. MTX has many potentially apoptotic actions [3,13,15] and NF-κB has many anti-apoptotic actions [36]. It, therefore, seems more likely that the fate of TNF-α and MTX-exposed macrophages is decided by the late effectors of apoptosis signaling, particularly at mitochondrial steps, that integrate and balance multiple converging inputs [43]. This requires better definition.

Macrophage NF-κB signaling is an attractive therapeutic target in RA because of roles in inflammation, differentiation and survival. However, NF-κB pathways are employed in diverse tissues, with broad biological consequences. Anti-TNF-α treatments suppress NF-κB activity in circumstances where it is promoted by TNF-α, such as in the synovial macrophage. Currently available NF-κB-suppressing small molecules, however, lack such cellular specificity. Glucocorticoids suppress macrophage NF-κB activity, but with poor specificity [21]. The proteosomal inhibitor, bortezomib, suppresses NF-κB signaling by preventing IκBα degradation and is reported to reduce synovial macrophage inflammatory activity in experimental murine collagen arthritis [44] but, again, is not specifically targeted to the macrophage. Thalidomide likewise suppresses clinical activity of systemic onset juvenile RA [45], and leads to monocyte apoptosis *in vitro *through actions on AKT-1 [46] and NF-κB signaling [47], but with the attendant threat of teratogenesis and other serious off-target effects. Developing macrophage-specific NF-κB suppressants may also have to contend with internal redundancy, with RelA and cRel able to serve similar functions for apoptosis suppression in haematopoietic cells [48].

## Conclusions

We were able to confirm that the potent NF-κB activator, TNF-α, counters MTX-induced apoptosis in primary murine macrophages. A less potent activator, RANKL, could not. Neither could the primary macrophage differentiation and survival factor, M-CSF, which we found did not activate NF-κB signaling. However, MTX did not suppress either constitutive or induced NF-κB signaling, ruling out a direct apoptotic action through NF-κB inhibition. These findings provide an additional explanation for the clinical complementarity between MTX and anti-TNF-α treatments for rheumatoid arthritis.

## Abbreviations

7-AAD: 7-aminoactinomycin D; AICAR: 5-aminoimidazole-4-carboxamide ribonucleotide; BAY: BAY11-7085; BMDM: bone marrow-derived macrophages; DISC: death-inducing signaling complex; DMEM: Dulbecco's Modified Eagle Medium; ERK: extracellular signal-regulated kinase; FCS: foetal calf serum; FLIP FLICE: inhibitory protein; GADD45β: growth arrest and DNA damage inducible protein; GM-CSF: Granulocyte macrophage-colony stimulating factor; IκB: inhibitor of NF-κB; IAP: inhibitors of apoptosis; IL-1, interleukin-1; JNK: jun N-terminal kinase; MAPK: mitogens-activated protein kinase; M-CSF: macrophage colony stimulating factor; Mn-SOD: manganese superoxide dismutase; MTT: 3-(4,5-Dimethylthiazol-2-yl)-2,5-diphenyltetrazolium bromide; MTX: methotrexate; NF-κB: nuclear factor-kappa-light-chain-enhancer of activated B cells; PAR: parthenolide; PDGF: platelet-derived growth factor; PI3K/AKT: phosphatidylinositol-3-kinase/protein kinase B; RANKL: receptor activator of NF-κB; RIP: receptor-interacting protein; TNF-α: tumor necrosis factor-alpha; TRAF TNF: receptor associated factor; VEGF: vascular endothelial growth factor; XIAP: X-linked inhibitor of apoptosis protein.

## Competing interests

The authors declare that they have no competing interests.

## Authors' contributions

SL performed the experiments and co-wrote the manuscript. DJ conceived of the study and co-wrote the manuscript. All authors were involved in the design of the study and interpretation of results.
